# Coupling between Properties of the Protein Shape and the Rate of Protein Folding

**DOI:** 10.1371/journal.pone.0006476

**Published:** 2009-08-03

**Authors:** Dmitry N. Ivankov, Natalya S. Bogatyreva, Michail Yu Lobanov, Oxana V. Galzitskaya

**Affiliations:** Institute of Protein Research, Russian Academy of Sciences, Pushchino, Russia; Stanford University, United States of America

## Abstract

There are several important questions on the coupling between properties of the protein shape and the rate of protein folding. We have studied a series of structural descriptors intended for describing protein shapes (the radius of gyration, the radius of cross-section, and the coefficient of compactness) and their possible connection with folding behavior, either rates of folding or the emergence of folding intermediates, and compared them with classical descriptors, protein chain length and contact order. It has been found that when a descriptor is normalized to eliminate the influence of the protein size (the radius of gyration normalized to the radius of gyration of a ball of equal volume, the coefficient of compactness defined as the ratio of the accessible surface area of a protein to that of an ideal ball of equal volume, and relative contact order) it completely looses its ability to predict folding rates. On the other hand, when a descriptor correlates well with protein size (the radius of cross-section and absolute contact order in our consideration) then it correlates well with the logarithm of folding rates and separates reasonably well two-state folders from multi-state ones. The critical control for the performance of new descriptors demonstrated that the radius of cross-section has a somewhat higher predictive power (the correlation coefficient is −0.74) than size alone (the correlation coefficient is −0.65). So, we have shown that the numerical descriptors of the overall shape-geometry of protein structures are one of the important determinants of the protein-folding rate and mechanism.

## Introduction

There is enormous diversity in the protein folding behavior from small proteins usually folding with simple two-state kinetics to large proteins usually folding with multi-state kinetics. Since misfolding, slow folding, and aggregation of proteins are responsible for many of the most devastating amyloid-related and other “conformational” diseases of the 21st century, it will be interesting and important to find new factors and parameters that correlate with protein folding rates.

There appear some general trends and correlations for the structural, thermodynamic, and kinetic properties of proteins [1]–[7]. The first comparison of a parameter with experimentally observed folding rates was made when it was shown that topology may be a critical determinant of two-state folding kinetics [3]. But the topology itself cannot explain the differences in the refolding rates for some proteins sharing the same fold (SH3 domains, cold shock proteins, fibronectin domains, proteins of the ferredoxin fold) [8]–[12].

A number of basic correlations between the protein size and folding rate have been suggested [1], [13], [14]. All of them point out that, as might be expected, the folding rate decreases with protein size, but suggest different scaling laws for this decrease. However, the current statistical analysis of protein folding data shows that all the suggested scalings, from –ln *L* to –*L* ^1/2^ and –*L* ^2/3^ correlate with the observed folding rates nearly equally: the correlation between folding rates and protein sizes is not large, about 65% [14]–[17]. It has been shown, that protein size *per se* determines folding rates of three-state folding proteins [5]. However, protein size, being the major determinant of the type of folding behavior, is not sufficient to determine the folding type of a protein since large proteins do not necessarily exhibit multi-state kinetics (for example, large helical protein Variable surface antigen VlsE folds with two-state kinetics [18]).

In the last years several models have been suggested to estimate the logarithm of the folding rate and structural parameters such as the contact order along with its modifications, the number of contacts, or the protein “effective length” [3], [7], [19]–[23]. These algorithms show a large magnitude of correlation coefficient between the folding rate and different structural features; however, they do not contribute to discriminating between two and multi-state kinetics.

Simultaneously, statistical and different machine-learning techniques were used to get high correlation with protein folding rates. Sometimes neural networks were used: for predicting folding rates of two-state proteins with known native structure, Dinner and Karplus [24] considered contact order and protein stability as the inputs to the neural network, while Zhang with collegues [25] used contact order, long-range order and total contact distance. A multiple regression technique was used for predicting protein folding rates from the protein secondary [26], [27] and primary structures [28]. Capriotti and Casadio [29] used a support vector machine for prediction of the protein folding kinetic order and rate from the known atomic structure. The multiple regression technique has allowed finding that proteins with two-state and multi-state kinetics have different rate-determining amino acids [30]. Although the amino acid composition may be one of the determinant factors for protein folding behavior, it does not make clear why the difference in intrinsic properties leads to a different folding type. On the contrary, it was demonstrated on a simple model that folding rates depend only on the topology of the native state but not on the sequence composition [31]. Overall, bioinformatical methods *per se* can not provide physical explanation of the obtained results.

The above rather conflicting results demonstrate that the theory of protein folding rate should be developed further. Therefore, the search for the factors affecting the protein folding process goes on.

There are several important questions on the coupling between properties of the protein shape and the rate of protein folding. Consideration of protein compactness specifically addresses the issue of why some proteins fold more rapidly than others. First, it has been shown that among proteins of the same size, α/β proteins have, on average, a greater number of contacts per residue due to their more compact (i.e., more “spherical”) structure [32], [33]. Next, we have suggested a relationship between the compactness expressed as the number of contacts per residue and folding rates (for 75 proteins for which both folding rates and tertiary structures are known): α-helical proteins have on average the fastest folding kinetics and the smallest number of contacts per residue (they are less compact than others), whereas α/β proteins have on average the slowest folding kinetics and the largest number of contacts (they are more compact than others) [33]. An explanation is that the expected surface of the boundary between folded and unfolded phases in the transition state for a more spherical protein is larger than for a non-spherical protein leading to a higher barrier and slower folding. Thus, the fact that α/β proteins are more spherical explains both the more average number of contacts per residue and the slower folding kinetics.

Since on average, the folding of multi-state proteins is slower than that of two-state ones, we should get further and define some numerical descriptors of the overall shape-geometry of protein structures to analyze their performance in predicting the folding behavior for a database of experimentally studied proteins. It turned out that parameters taking into account both the size and characteristics of the protein shape correlate well with the logarithm of the folding rate. We demonstrated that the radius of cross-section is a highly sensitive parameter that can be used to predict the protein folding rates and their possible mechanism of folding.

## Methods

### Data Sets

We have considered 84 single-domain proteins or separate domains of multi-domain proteins for which both folding rates and tertiary structures are known [33], [34]. Among them 26 proteins exhibit multi-state kinetics and 58 proteins exhibit two-state kinetics (see http://phys.protres.ru/resources/compact.html).

We have selected single-domain proteins or separate domains of multi-domain proteins having from 51 to 350 residues with less than 25% sequence identity belonging to classes ‘a’ (all-α proteins), ‘b’ (all-β proteins), ‘c’ (α/β proteins), and ‘d’ (α+β proteins), according to the SCOP classification [35], release 1.65. The obtained database includes 3413 proteins: 702 all-α proteins, 868 all-β proteins, 914 α/β proteins, and 929 α+β proteins.

### Calculation of protein structure compactness

We have calculated the solvent-accessible surface area 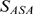 and volume 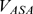 surrounded by this surface, and also volume 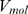 enclosed by the protein molecular surface 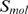 for each protein considered. We accomplished the calculations using the YASARA program [36] [http://yasara.org], setting the radius of a probe molecule to be 1.4 Å.

We consider a series of structural descriptors intended to describe protein shapes:


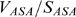
 is proportional to the average radius of the minimal cross-section in the center of protein molecule (for short, sometimes we will use the name “radius of cross-section” for this parameter). Among different geometric bodies of equal volumes, this ratio should be maximal for a sphere. This value has the dimension of length and depends on the protein size.


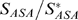
 which Zehfus and Rose called the “coefficient of compactness” [37] is the ratio of the accessible surface area of protein 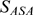 to the surface area of sphere 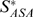 of equal volume *V_ASA_* as that of the protein (for sphere this ratio is 1).

*Radius of gyration.* If we consider atoms as points in the 3D space, the radius of gyration *R_g_* of a protein is calculated as:
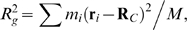
(1)where *m_i_* is the mass of the *i*-th atom, **r** *_i_* is its Cartesian coordinates, *M* is the mass of the protein, and **R** *_C_* is the coordinate vector of the mass center of the protein calculated as follows:
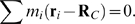
(2)Since PDB files with protein structures often lack hydrogen atoms, then in eq. (1) only non-hydrogen atoms should be taken into account, and *M* is the net mass of non-hydrogen atoms.

For calculation of the normalized radius of gyration we computed the radius of gyration of a ball of uniform density and of equal volume as that of the considered protein according to the following equation:
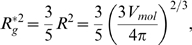
(3)where *V_mol_* is the volume enclosed within the protein molecular surface. Note that the radii of gyration 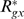, 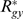, and 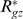 of a ball of uniform density corresponding to the rotations around Cartesian axes *x*, *y*, and *z* going through the mass center are calculated as 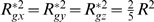. The factor 3/5 in eq. (3) instead of 2/5 in the latter equation is explained by the fact that there are many axes a protein can be rotated around; in this case the radius of gyration “averaged” over all possible rotations is calculated as 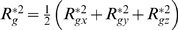 [38].

## Results

### Different measures of compactness in protein folding study

After more than 30 years we return to the measure of compactness which was suggested by Wetlaufer [39]. Wetlaufer measured the compactness of a protein (or a protein part) by the use of its surface to volume ratio, normalized by the surface to volume ratio for a ball of equal volume. The parameter introduced by Wetlaufer is equal to the protein surface normalized by the surface of a ball of equal volume. In analogy to hydrodynamic frictional ratios, this relative surface area should form a numerical scale on which the more compact structure will have smaller values [39]. In a number of modifications, this parameter appeared later as “roughness index” [40], “globularity index” [41], “coefficient of compactness” [37], and “compactness” [42]. We use here the name “coefficient of compactness” [37] and its definition as the accessible surface area of a protein normalized by the surface area of a ball of equal volume (minimum possible surface area).

The overall shape of an object becomes the factor that determines compactness if packing efficiency is uniform [37]. Our analysis of 3413 protein structures having from 51 to 350 residues revealed that packing efficiency, indeed, is the same for proteins from different structural classes evidenced by the molecular volume per atom (for all-α proteins – 18.520±0.010 Å^3^, for all-β proteins −18.577±0.009 Å^3^, for α/β proteins −18.618±0.007 Å^3^, and for α+β proteins −18.598±0.009 Å^3^).

In addition to the coefficient of compactness, we have used other measures of compactness in our work: the radius of gyration and the radius of cross-section (see Methods).

**Figure 1:**
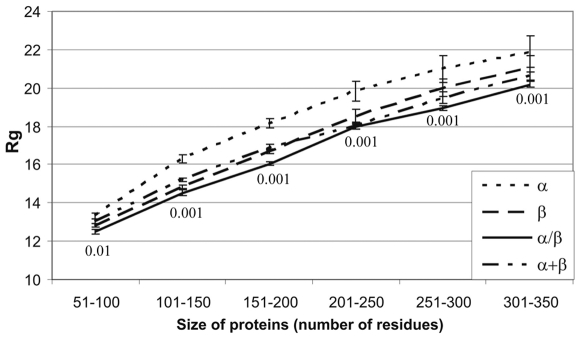
Dependence of radius of gyration on the number of residues in proteins for general structural classes. Average errors are given. Structural classes are indicated. Below each point corresponding to α/β proteins, the probability that the observed difference in average values is occasional is given. The probabilities were calculated with Student's t-test (probabilities, α/β vs. α proteins is shown).

First, we have inspected the properties of unnormalized radius of gyration as a measure of compactness describing the shape of the protein globule. The statistical analysis of radii of gyration for 3413 protein structures from four general structural classes (all-α, all-β, α/β, α+β) demonstrates that each class of proteins has its own class-specific radius of gyration, which determines the shape of protein structures: α-proteins have the largest radius of gyration while α/β-proteins have the least radius of gyration (see Figure 1). This shows that α-proteins are less spherical, and α/β proteins are most spherical among proteins of four structural classes. This is similar to the result obtained earlier for other measures of compactness, namely, for the coefficient of compactness 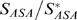 and the number of contacts per residue [33].

**Figure 2:**
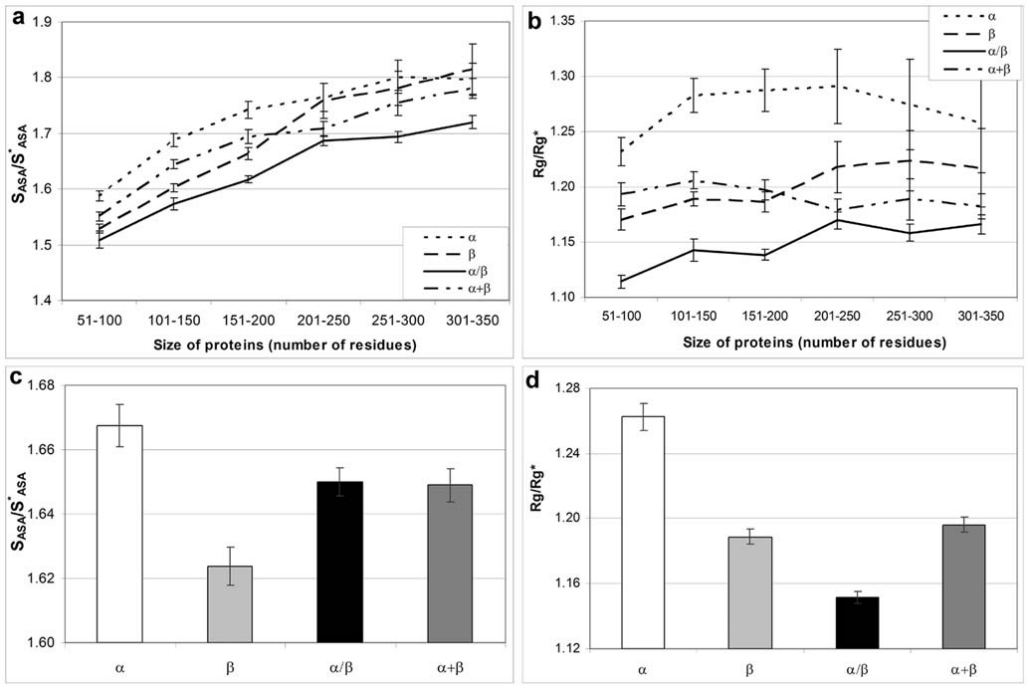
Average coefficient of compactness and average normalized radius of gyration for proteins from different structural classes. (A) Dependence of average coefficient of compactness on the number of protein residues for general structural classes. (B) Dependence of average normalized radius of gyration on the number of protein residues for general structural classes. (C) Average coefficient of compactness for proteins from different structural classes. (D) Average normalized radius of gyration for proteins from different structural classes. In each panel average errors are given. Structural classes are indicated.

The clearly seen dependence of the radius of gyration on the protein length (Figure 1) is not convenient for an analysis since it forces us to use several size windows. From the other measures of compactness, the number of contacts per residue and the radius of cross-section, similar to the radius of gyration, are expected to depend on the protein size and they do so [33]. The only measure that could be independent of the protein size is the coefficient of compactness, since it is a normalized parameter. Though, it also depends on the protein size (Figure 2a), and this dependence is crucial since consideration of all proteins taken together (i.e. without sorting them according to their size) changes the result dramatically, namely: α/β proteins are erroneously classified as proteins with intermediate compactness (Figure 2c) while actually they have the highest compactness as judged by the analysis using different window sizes (Figure 2a). Such a situation is a result of a different number of proteins in each region of size. Therefore, the average value over six regions does not necessarily coincide with the average over all proteins without dividing them into regions. Thus, to avoid the dependence on length, we introduce a new measure of compactness as a protein radius of gyration normalized to the radius of gyration of a ball with equal volume. It turns out that this measure does not depend on the protein size (Figure 2b), and as a consequence, the results of averaging over all proteins are the same as those for the window-sized analysis, namely, α/β proteins are the most compact, i.e. have a more spherical shape than the others (Figure 2b,d).

The results for different measures of compactness and for different structural classes are presented in Table 1.

**Table 1:** Average values of different measures of compactness for proteins from four general structural classes given for different size windows and for whole classes.

	All	51–100	101–150	151–200	201–250	251–300	301–350
Average value of radius of gyration, *Rg*, Å							
α	15.93±0.15	13.3±0.2	16.3±0.2	18.2±0.3	19.9±0.5	21.0±0.7	22.0±0.8
β	15.44±0.10	12.8±0.1	14.8±0.1	16.7±0.1	18.5±0.4	20.0±0.5	21.2±0.7
α/β	17.20±0.09	12.1±0.4	14.5±0.2	16.1±0.1	17.9±0.1	19.0±0.1	20.2±0.2
α+β	15.77±0.09	13.1±0.1	15.2±0.1	16.9±0.1	18.0±0.2	19.5±0.3	20.6±0.2
Average value of normalized radius of gyration, Rg/Rg^*^							
α	1.263±0.008	1.23±0.01	1.28±0.02	1.29±0.02	1.29±0.03	1.27±0.04	1.26±0.05
β	1.189±0.005	1.17±0.01	1.19±0.01	1.19±0.01	1.22±0.02	1.23±0.03	1.22±0.04
α/β	1.154±0.004	1.08±0.04	1.14±0.01	1.14±0.01	1.17±0.01	1.16±0.01	1.17±0.01
α+β	1.196±0.004	1.19±0.01	1.21±0.01	1.20±0.01	1.18±0.01	1.19±0.02	1.18±0.01
Average value of radius of cross-section, *V_ASA_/S_ASA_*, Å							
α	3.749±0.017	3.44±0.02	3.76±0.02	4.00±0.03	4.27±0.05	4.45±0.06	4.67±0.07
β	3.930±0.014	3.58±0.02	3.85±0.02	4.15±0.02	4.26±0.05	4.48±0.06	4.64±0.09
α/β	4.388±0.014	3.67±0.04	3.97±0.02	4.25±0.01	4.43±0.02	4.68±0.02	4.85±0.03
α+β	3.934±0.015	3.53±0.02	3.81±0.01	4.10±0.02	4.37±0.03	4.55±0.05	4.72±0.04
Average value of coefficient of compactness, S_ASA_/S^*^_ASA_							
α	1.667±0.007	1.59±0.01	1.69±0.01	1.74±0.02	1.76±0.03	1.80±0.03	1.80±0.03
β	1.624±0.006	1.53±0.01	1.60±0.01	1.66±0.01	1.76±0.03	1.78±0.03	1.82±0.05
α/β	1.649±0.004	1.51±0.02	1.57±0.01	1.62±0.01	1.69±0.01	1.69±0.01	1.72±0.01
α+β	1.649±0.005	1.55±0.01	1.64±0.01	1.69±0.01	1.71±0.01	1.75±0.02	1.78±0.02

### Relationship between the parameters describing the protein globule shape and the protein folding rate

Structural descriptors intended to describe protein shapes have been divided into four groups: parameters of compactness connected with the radius of cross-section, parameters of compactness which are not connected with the protein size, other parameters depending on the protein size, and other parameters not depending on the protein size.

According to analytical theory of protein folding based on the nucleation model [1], the logarithm of the folding rate should be proportional to the surface of the boundary between two phases (folded and unfolded) in the transition state. However, parameters of compactness considered above are closely related to this boundary, so the surface of the boundary can be roughly estimated from the protein native structure. For example, (*V_ASA_/S_ASA_*)^2^ is proportional to the square of the minimal cross-section drawn through the center of a protein molecule, while *Rg* ^2^ is roughly proportional to the surface of the maximal cross-section. We have also considered parameters *V_ASA_/Rg* and *L* ^2/3^; the latter is the size of the average cross-section drawn through the protein center for a spherical protein since it does not take into account the protein shape. Correlation coefficients between the considered parameters and the logarithm of folding rates for 84 proteins have been calculated and are given in Table 2. One can see that these parameters can predict the folding rate (the correlation coefficient is larger than 0.7 in most cases). It should be mentioned that the correlation of protein folding rates with *Rg* ^2^ (proportional to the surface of maximal cross-section) is worse than with (*V_ASA_/S_ASA_*)^2^ (proportional to the surface of minimal cross-section drawn through the center of the protein molecule). This is quite predictable since a protein prefers to fold through the transition state with the least surface of the boundary between two phases.

**Table 2:** Correlation coefficients between logarithms of folding rates in water and different parameters of protein structure.

	ln *k_F_ 84 proteins*	ln *k_F_^mult^ 26 proteins*	ln *k_F_^two^ 58 proteins*
Parameters of compactness connected with size of cross-section (they depend on protein size)			
*Rg^2^*	−0.53±0.08	−0.72±0.09	−0.32±0.12
*L^2/3^*	−0.69±0.06	−0.80±0.07	−0.47±0.10
*(V_ASA_/S_ASA_)^2^*	−0.73±0.05	−0.76±0.08	−0.57±0.09
*V_ASA_/Rg*	−0.72±0.05	−0.81±0.07	−0.53±0.09
Parameters of compactness normalized to exclude dependence on protein size (they are expected to be independent of protein size)			
S_ASA_/S*_ASA_	−0.33±0.10	−0.48±0.15	−0.19±0.13
Rg/Rg*	0.23±0.10	−0.01±0.20	0.20±0.13
Parameters of protein size and average size of protein loop			
*L*	−0.65±0.06	−0.78±0.08	−0.42±0.11
*L^1/2^*	−0.70±0.06	−0.81±0.07	−0.50±0.10
ln *L*	−0.71±0.05	−0.82±0.06	−0.55±0.09
*AbsCO*	−0.77±0.04	−0.78±0.08	−0.71±0.06
Relative contact order – parameter of average size of protein loop normalized to exclude dependence on protein size			
*CO*	−0.01±0.11	0.25±0.18	−0.41±0.11

**Figure 3:**
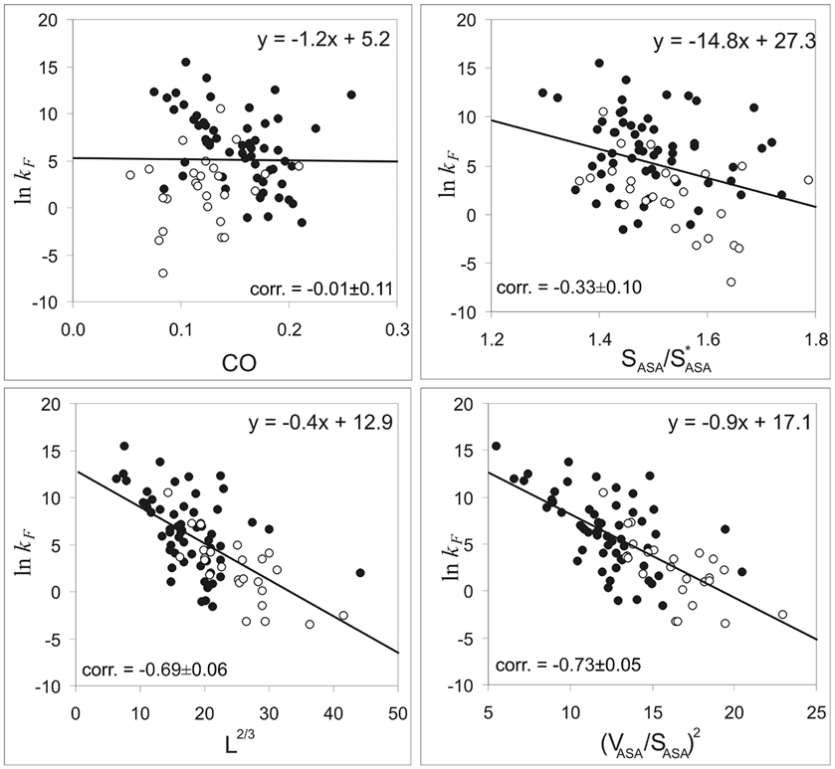
Dependence of the logarithm of the folding rate in water for multi-state and two-state folders on several investigated parameters. Black circles correspond to two-state folders and open circles correspond to multi-states folders.

Parameters normalized for excluding the dependence on protein size, whether they describe compactness (Rg/Rg^*^, S_ASA_/S^*^_ASA_) or average length of loop (*CO*), correlate worse with the logarithm of folding rate (see Table 2 and Figure 3a,b). It turned out that when a descriptor is normalized to eliminate the influence of protein size, it completely looses its ability to predict folding rates. On the other hand, when a descriptor correlates well with protein size (the radius of cross-section and absolute contact order in our case) it would correlate well with the logarithm of folding rates (see Table 2, Figure 3c,d). It should be stressed that among normalized parameters, S_ASA_/S^*^_ASA_ which slightly depends on protein size (Figure 2a) correlates somewhat better with folding rates than Rg/Rg^*^, which is independent of the protein size (Figure 2b).

**Figure 4:**
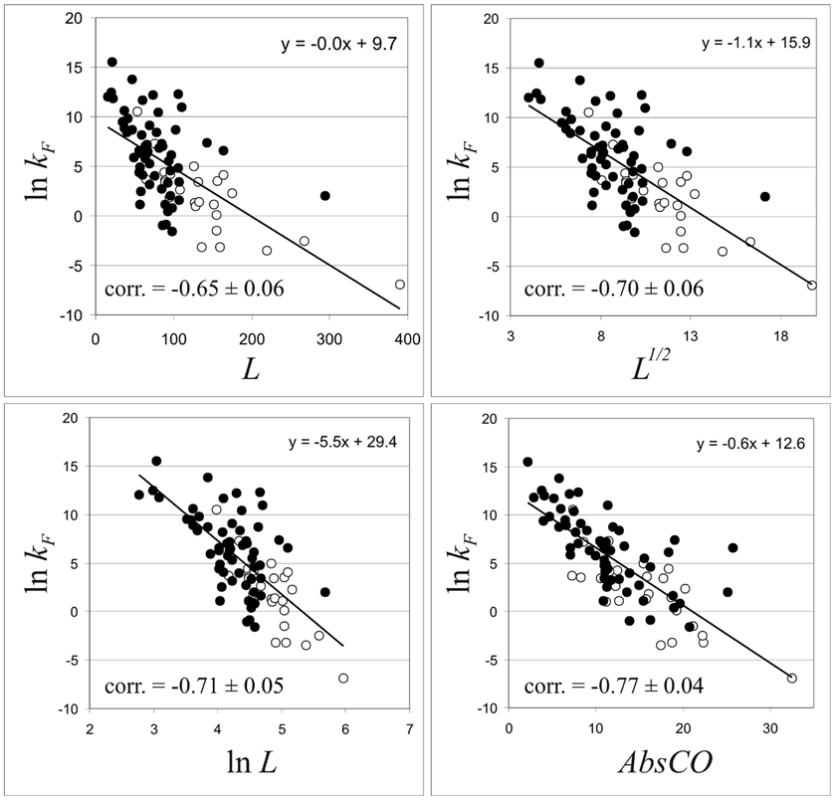
Dependence of the logarithm of the folding rate in water for multi-state and two-state folders on the protein size in various colors (*L*, *L* ^1/2^, ln *L*) and absolute contact order. Black circles correspond to two-state folders and open circles correspond to multi-states folders.

Since all measures of the size of the cross-section ((*V_ASA_/S_ASA_*)^2^ and *L* ^2/3^) are highly dependent on the protein size, we have controlled critically the performance of the new descriptors with protein size. The correlations of protein size in the various colors (*L*, ln *L*, *L* ^1/2^) with the logarithm of folding rates have been calculated (see Table 2 and Figure 4). One can see that not normalized parameters of cross-section, (*V_ASA_/S_ASA_*)^2^ and *L* ^2/3^ have a slightly higher predictive power than the size alone [r = −0.73 for (*V_ASA_/S_ASA_*)^2^, the P-value associated with this correlation, P = 0.00002, is extremely low, suggesting that the observed correlation is highly improbable to have arisen by chance; r = −0.69 for *L* ^2/3^, P = 0.0002. r = −0.65 for *L*, P = 0.0009.]. However, pure theoretical parameter *L* ^2/3^ is statistically indistinguishable from *L* (see errors of correlation in Table 2). On the other hand, the new descriptor (*V_ASA_/S_ASA_*)^2^ is statistically distinguishable from *L*, i.e. is not just a complicated reformulation of size scaling effects, thus, providing some information on the compactness of the protein globule. It should be noted, that ln *L* and *L* ^1/2^ have almost the same performance [r = −0.71 for ln *L*, the P-value associated with this correlation, P = 0.00004; r = −0.70 for *L* ^1/2^, P = 0.00004] as *V_ASA_/S_ASA_*; however, ln *L* does not ensue from any physical theory. At the same time one can see that such a parameter as *AbsCO* has a slightly higher predictive power than the radius of cross-section (*V_ASA_/S_ASA_*)^2^ [r = −0.77 for *AbsCO*; the P-value associated with this correlation, P = 0.000001, is extremely low, suggesting that the observed correlation is highly improbable to have arisen by chance. r = −0.73 for (*V_ASA_/S_ASA_*)^2^; the P-value associated with this correlation, P = 0.00002].

We performed also a very useful analysis which represents the connection between the correlation coefficient and the shape of a cloud of points (see Table S1). One can see that the forms of ellipsoids are different for correlation coefficients −0.77, −0.73, −0.69 and −0.65: the relations of short to long axes are 0.26, 0.30, 0.35, and 0.41, respectively. In an ideal case when correlation coefficient tends to one, the relation of axes tends to zero.

Considering the correlations of protein size in various colors (ln *L*, *L* ^1/2^, *L* ^2/3^) with the logarithm of folding rates one can summarize up that, similar to ln *L* and *L* ^1/2^, not normalized parameters of cross-section, (*V_ASA_/S_ASA_*)^2^ (reflecting the shape of the protein globule) and *L* ^2/3^, work slightly better than *L*. This shows that the improvement over protein length can be interpreted in different ways, one of which is the proposed here importance of cross-section in determining of protein folding kinetics.

### Comparison of different parameters describing the shape of protein globules for two-state and multi-state folders

On average, the folding of multi-state proteins is slower than that of two-state ones. At the same time, slow folding proteins are more spherical (compact) than fast folding ones [43]. Therefore, it is natural to expect that multi-state proteins are more compact than two-state folders, and the larger boundary expected for more spherical proteins results in a higher free-energy barrier for folding.

For two-state and multi-state proteins, we have averaged the coefficient of compactness for accessible and molecular surfaces, the radius of cross-section, the normalized radius of gyration, the absolute contact order and the logarithm of folding rates in order to compare them. One can see that multi-state proteins are indeed more spherical and rough than proteins with two-state kinetics as judged by all considered descriptors of the protein shape (see Table 3) except for 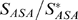 that has a slight dependence on the protein size (see Fig. 2a). If to consider proteins from some size range (50–100 or 101–150 a.a. residues, see Table 4) one can see that 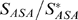 is smaller for multi-state proteins than for two-state ones. The differences are more distinct for other parameters if proteins are divided into groups by size range (see Table 4). From Table 4 one can see that proteins with multi-state kinetics, on average, are more spherical than proteins with two-state kinetics; this is still true for normalized parameters. As concern coefficient of compactness, then from Fig. 2a one can see that it grows with protein size. That is, it can be concluded that longer polypeptides are more likely to both fold via non-two-state mechanisms and to be more spherical.

**Table 3:** Average values of normalized radius of gyration, coefficient of compactness for accessible and molecular surface, radius of cross-section, absolute contact order and logarithms of in-water folding rates for two- and multi-state folders.

Proteins	All, 84 proteins	Multi-state, 26 proteins	Two-state, 58 proteins
Rg/Rg*	1.18±0.01	1.14±0.01	1.19±0.02
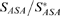	1.51±0.01	1.53±0.02	1.50±0.01
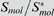	2.25±0.03	2.45±0.04	2.16±0.04
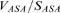	3.65±0.05	4.07±0.08	3.46±0.05
*AbsCO*	12.56±0.63	15.60±1.13	11.20±0.69
ln *k_F_*	5.1±0.5	2.0±0.8	6.4±0.5

**Table 4:** Average values of different measures of compactness and logarithms of in-water folding rates for two- and multi-state folders for the considered size range.

Folding kinetics	50–100		101–151	
Size range, residues	Two-state	Multi-state	Two-state	Multi-state
Number of proteins	36	9	7	8
Rg/Rg*	1.16±0.01	1.13±0.01	1.30±0.04	1.13±0.01
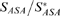	1.50±0.01	1.45±0.02	1.60±0.03	1.52±0.01
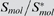	2.18±0.03	2.22±0.05	2.41±0.06	2.45±0.05
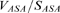	3.54±0.03	3.75±0.05	3.76±0.05	4.14±0.07
*AbsCO*	12.21±0.62	12.00±1.34	13.00±1.50	15.30±1.06
ln *k_F_*	4.98±0.57	5.12±0.84	7.03±1.40	1.58±0.84

**Figure 5:**
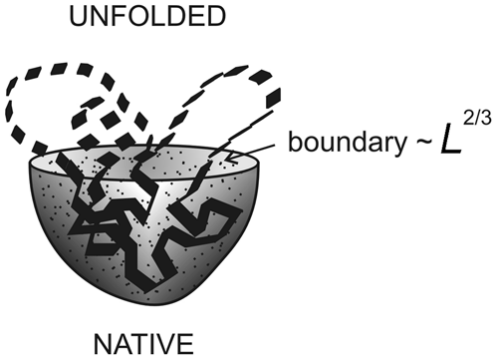
Transient semi-unfolded (and semi-folded) state of protein. The unfolded part is shown by dashed lines, the folded structure is shown by solid lines. Unfolded closed loops protruding from the folded part (the nucleus) create an additional surface tension.

We suggest the following explanation: the slower folding for multi-state proteins of the same size can be explained by their more spherical structure so the expected surface of the boundary between folded and unfolded phases in the transition state [16] for a more spherical protein is larger than that for a non-spherical protein (see Figure 5).

## Discussion

One of the first analytical theories of protein folding for one-domain globular proteins was Finkelstein-Badretdinov's theory based on the capillarity model [1]. In the frame of this model, at the point of thermodynamic equilibrium between folded and unfolded states the rate of protein folding depends on the size of the boundary between two phases (folded and unfolded), and unfolding closed loops protruding from the folded part, the nucleus, create an additional surface tension, which results in deceleration of the protein folding. Since the boundary between two phases depends on the number of amino acids for spherical globules as *L* ^2/3^, then the folding rate at the point of equilibrium between native and unfolded states should depend on the number of amino acids in a similar way 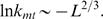



In this work we demonstrate that the radius of cross-section is a highly sensitive parameter that can be used to predict the protein folding rates and their possible mechanism of folding. Both parameters of cross-section, *L* ^2/3^ and, especially (*V_ASA_/S_ASA_*)^2^ which reflects the shape of the protein globule, work slightly better than *L*.

Comparison of proteins having similar native topologies is an important test for understanding fundamental aspects of the protein folding process. One of the known families of homologous proteins is that of fibronectin type III modules (FNIII). Though the proteins are homologous, the ninth module folds several hundred times slower than the tenth in the absence of a denaturant [44]. The authors who studied these proteins explained the different folding kinetics of the two modules by a large difference in their thermodynamic stability. The analysis done in this study can provide an additional explanation for this difference: despite structural similarities, the coefficient of compactness of the ninth module (1.47) is less than that of the tenth module (1.53). The difference for the radius of cross-section demonstrates the same trend in compactness (3.75 and 3.62, for the 9^th^ and 10^th^ modules, correspondingly). Both modules fold via two-state kinetics [44] (it should be mentioned that the use of a more strong denaturant reveals the population of intermediates in the faster folding of the 10^th^ module [45]), and the relatively slow folding of the ninth module is not due to the occurrence of a slowly folding on-pathway intermediate [46]. Different refolding rates of the set of homologous proteins can be explained by different compactness of the protein structures.

Another interesting example is the folding of cold shock proteins. The absence of correlation between the thermodynamic stability and folding rate, as observed for cold shock proteins, indicates that proteins with a more stable folded state do not necessarily fold faster. At the same time, Cold shock proteins B are less compact (average compactness for three proteins is 1.50) than Cold shock proteins A (compactness is 1.43), and the folding of Cold shock proteins B is faster [11]. The difference for the radius of cross-section demonstrates the same trend in compactness (3.40 Å and 3.54 Å for Cold shock proteins B and Cold shock protein A, correspondingly). However, among three Cold shock proteins B the differences in folding rates are too small to be explained by the differences in compactness.

Thus, more spherical proteins indeed fold more slowly than proteins with an elongated shape. Under equal conditions, a more spherical, more compact protein is not able to avoid the large boundary between two phases (folded and unfolded) in the transition state independent of the folding pathway. A more elongated, less compact protein has a possibility to choose such a pathway of folding in which the protein folding goes through the small boundary between two phases and consequently through a rather low free energy barrier of folding.

**Figure 6:**
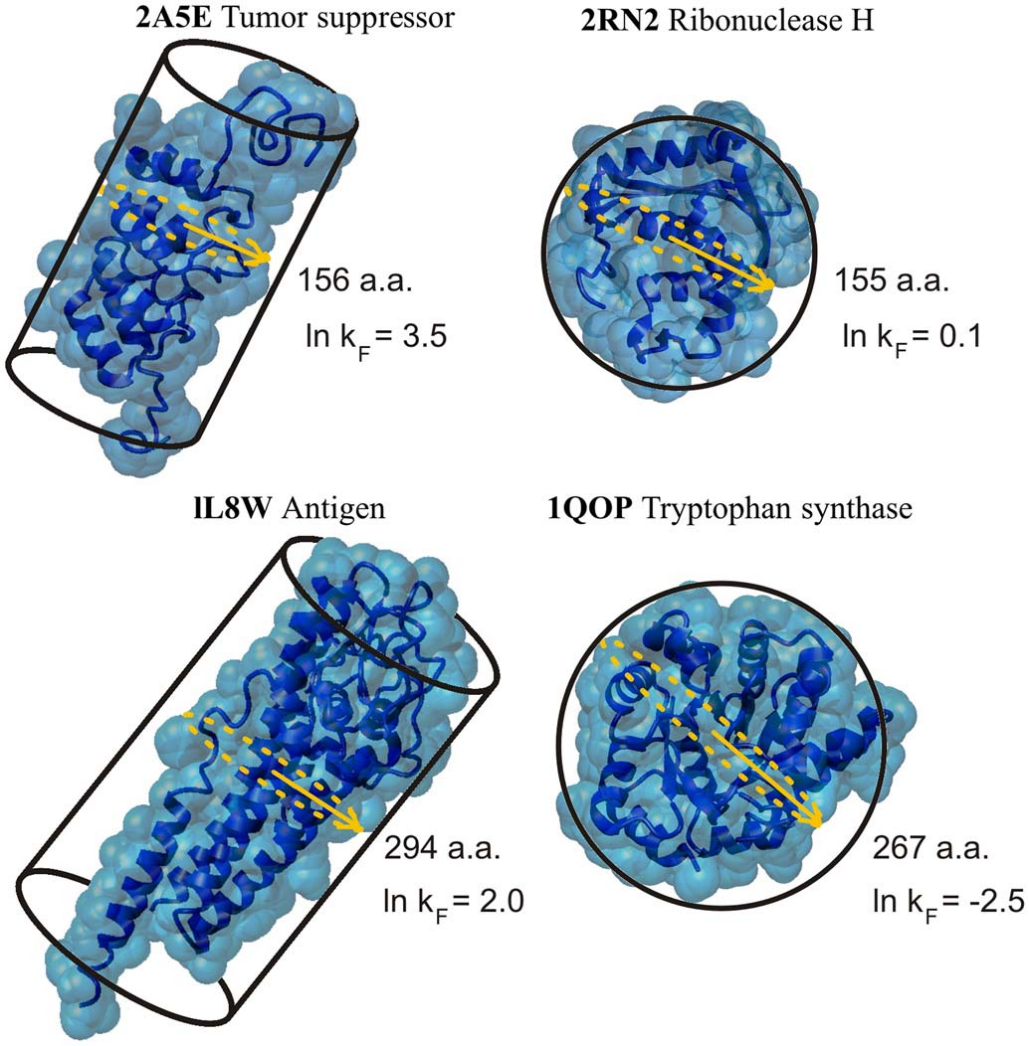
Influence of minimal protein globule cross-section on the folding rate. More elongated, cylinder-like proteins VlsE (PDB entry 1L8W, 294 residues) and p16 (PDB entry 2A5E, 156 residues) having small cross-section fold more rapidly than more spherical proteins RNase HI (PDB entries 2RN2, 155 residues) and Tryptophan-synthase α-subunit (PDB entry 1QOP, 267 residues) having large cross-section.

Most striking examples of protein folding illustrating this scenario are as follows: the variable surface antigen VlsE (PDB entry 1L8W) is less spherical than a protein with a similar number of amino acid residues which has multi-state kinetics (Tryptophan synthase α subunit, PDB entry 1QOP, see Figure 5 and Tables 1 and 2 at the http://phys.protres.ru/resources/compact.html). VlsE does not obey the contact-order correlation which can be explained in terms of the entropy cost of the size of loops and/or the ordering of residues between contacting residues [18]. Another example is a pair of proteins RNase HI and p16 (PDB entries 2RN2 and 2A5E, respectively, see Figure 6). From our analysis we can conclude that the barrier height for folding of large proteins is defined by the size of the boundary surface between folded and unfolded phases in the transition state.

The prediction of protein folding rates has its own practical value due to the fact that aggregation directly depends on the rate of protein folding. It is worthwhile to underline that the results of our analysis allow us to suggest additional parameters for determining the folding type of a protein.

As a result of our analysis, two conclusions can be made. First, similar to some other papers emphasizing the influence of protein chain length on folding rate [1], [5], [13], [14], [19], [47], we have found the same effect on our set of protein shape parameters: in order to predict protein folding rates, a parameter should correlate well with the protein chain length. As a very illustrative example of such kind was observed with relative contact order (the normalized parameter, which has poor correlation with the logarithm of protein folding rates, see Table 2) and absolute contact order, which includes both the size of protein and the average length of loops and correlates well with the logarithm of protein folding rates [3], [19]. Second, we have found that the protein shape expressed by different parameters could be an important determinant of the protein folding kinetics and protein folding type. The more spherical is the protein the slower folding it exhibits. Proteins with multi-state kinetics, on average, are more spherical than proteins with two-state kinetics. The barrier height for folding of large proteins is defined by the size of the boundary between folded and unfolded phases in the transition state. This boundary is larger for a spherical shape of the protein globule than for the elongated one.

## Supporting Information

Table S1 Connection between the correlation coefficient and the shape of a cloud of points
(0.23 MB DOC)
