# Supplementary material for: Coupling between Properties of the Protein Shape and the Rate of Protein Folding
Source: PLoS One. 2009 Aug 3;4(8):e6476. doi: 10.1371/journal.pone.0006476 (PMC2714458; doi:10.1371/journal.pone.0006476)
Supplement: Table S1 — Connection between the correlation coefficient and the shape of a cloud of points (0.23 MB DOC) [file pone.0006476.s001.doc]

**Table S1.** Connection between the correlation coefficient and the shape of a cloud of points

|  | ln *k_F_*  *84 proteins* | ln *k_F_^multi^*  *26 proteins* | ln *k_F_^two^*  *58 proteins* |
| --- | --- | --- | --- |
| Parameters of compactness connected with size of cross-section (they depend on protein size) | | | |
| *Rg^2^* | -0.53±0.08  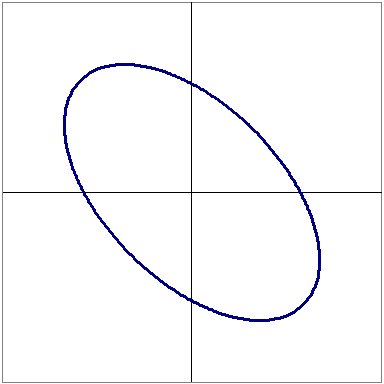 | -0.72±0.09  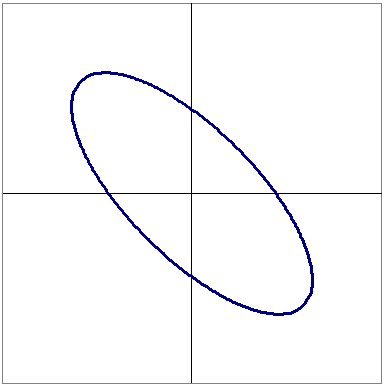 | -0.32±0.12  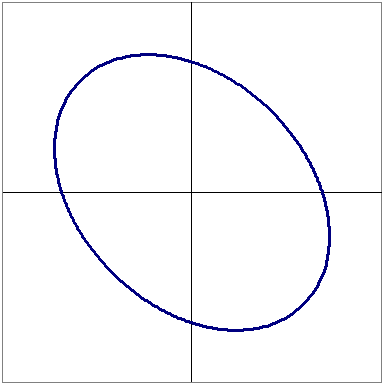 |
| *L^2/3^* | -0.69±0.06  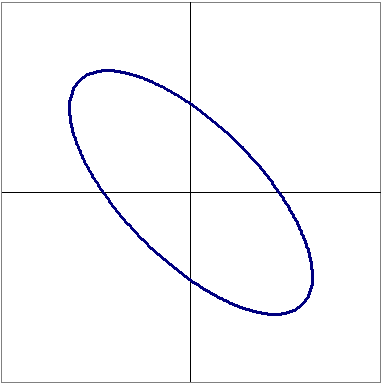 | -0.80±0.07  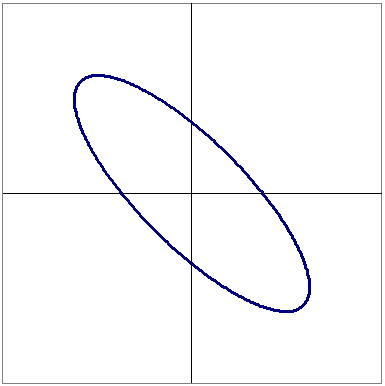 | -0.47±0.10  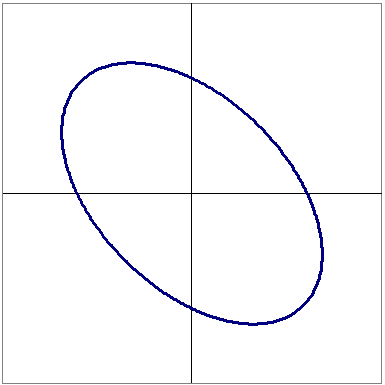 |
| *(V_ASA_/S_ASA_)^2^* | -0.73±0.05  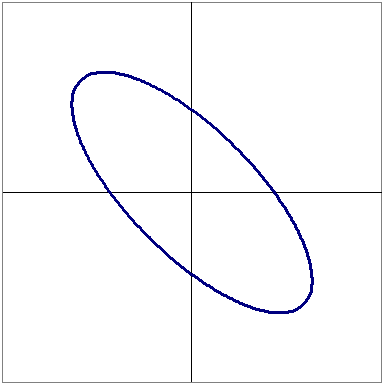 | -0.76±0.08  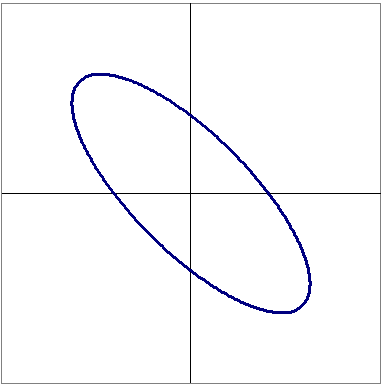 | -0.57±0.09  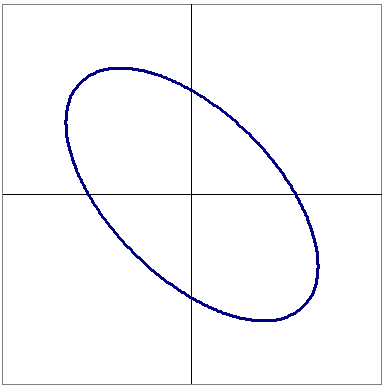 |
| *V_ASA_/Rg* | -0.72±0.05  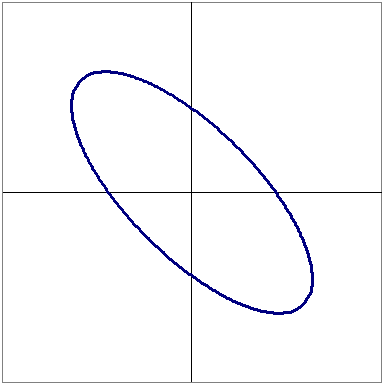 | -0.81±0.07  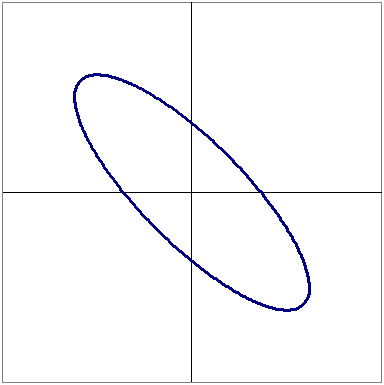 | -0.53±0.09  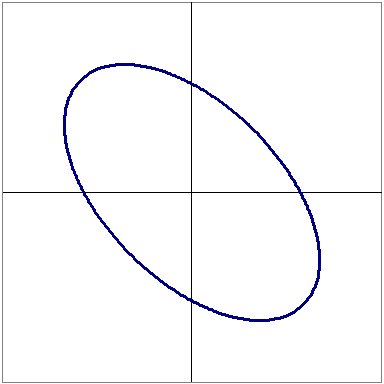 |
| Parameters of compactness normalized to exclude dependence on protein size (they are expected to be independent of protein size) | | | |
| *S_ASA_/S*_ASA_* | -0.33±0.10  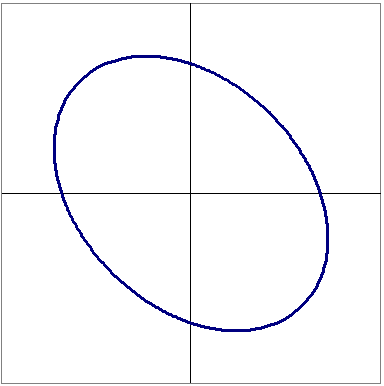 | -0.48±0.15  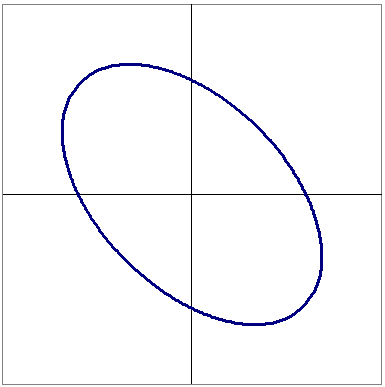 | -0.19±0.13  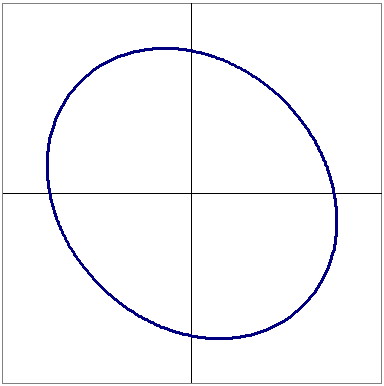 |
| *Rg/Rg** | 0.23±0.10  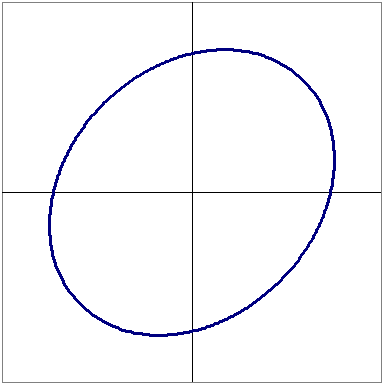 | -0.01±0.20  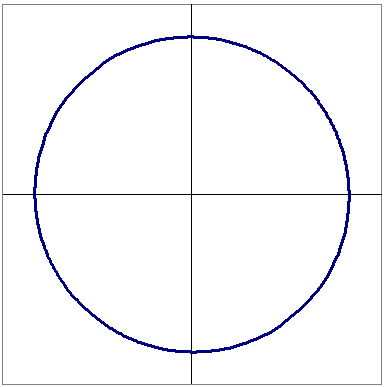 | 0.20±0.13  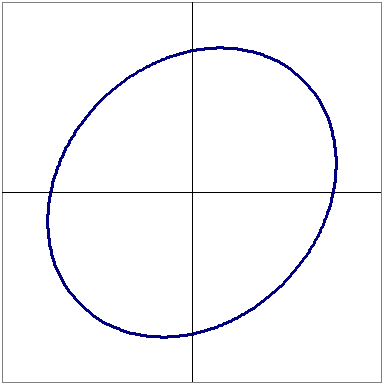 |
| Parameters of protein size and average size of protein loop | | | |
| *L* | -0.65±0.06  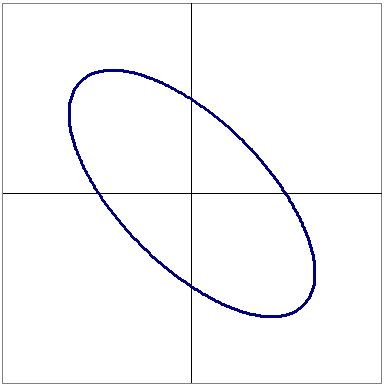 | -0.78±0.08  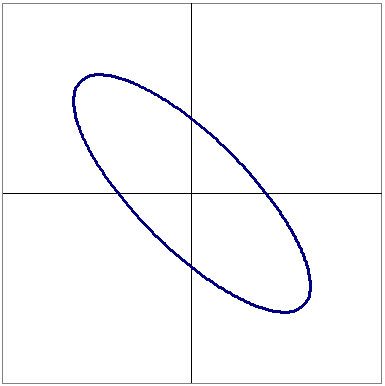 | -0.42±0.11  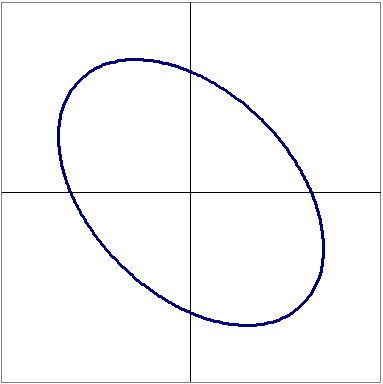 |
| *L^1/2^* | -0.70±0.06  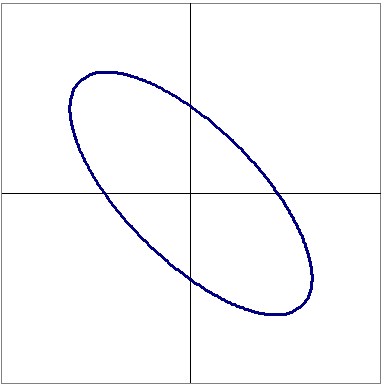 | -0.81±0.07  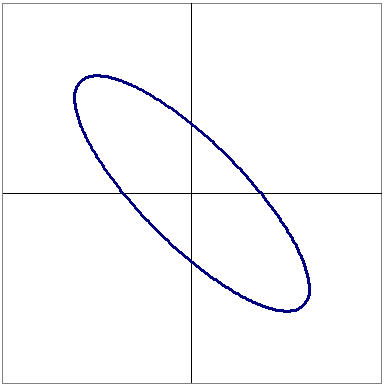 | -0.50±0.10  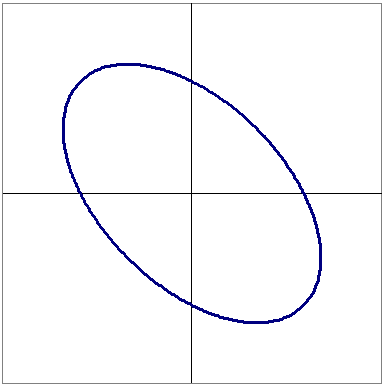 |
| ln *L* | -0.71±0.05  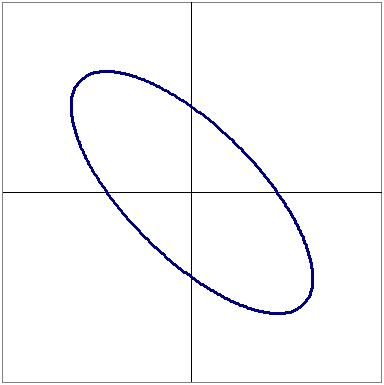 | -0.82±0.06  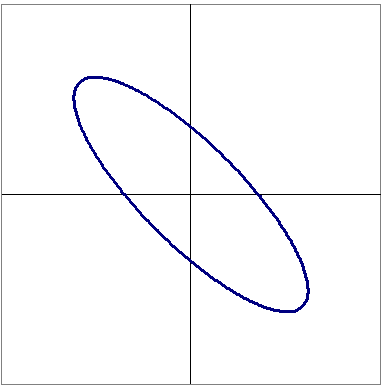 | -0.55±0.09  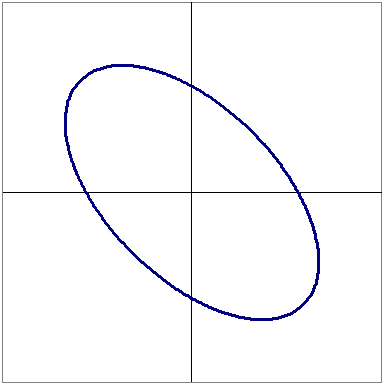 |
| *AbsCO* | -0.77±0.04  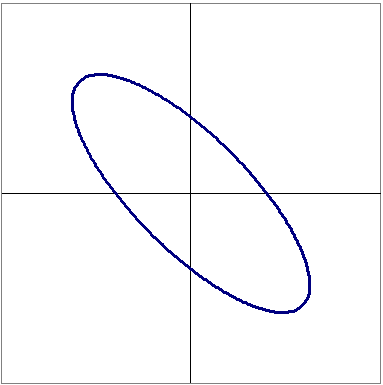 | -0.78±0.08  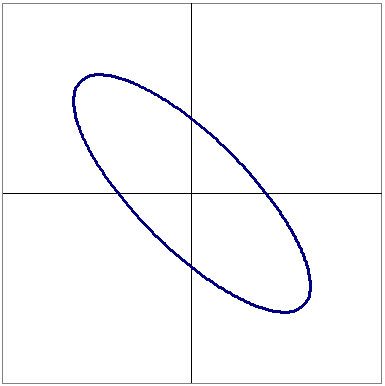 | -0.71±0.06  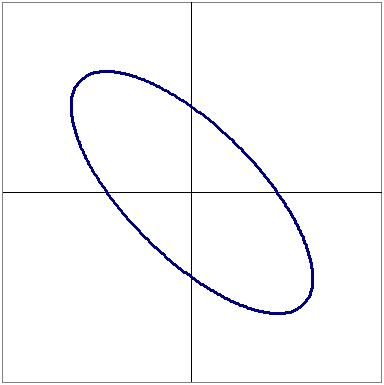 |
| Relative contact order – parameter of average size of protein loop normalized to exclude dependence on protein size | | | |
| *CO* | -0.01±0.11  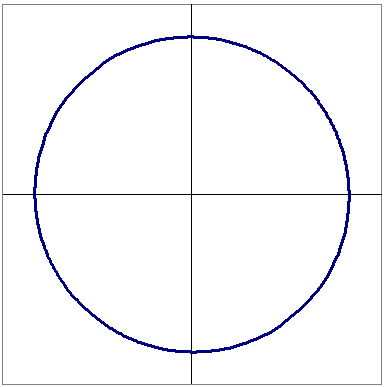 | 0.25±0.18  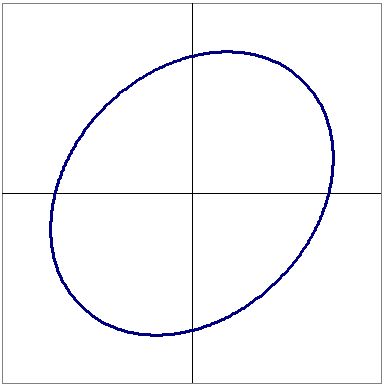 | -0.41±0.11  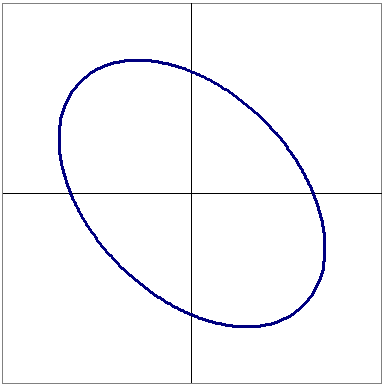 |

The relation of short to long axes is calculated according to equation:

K=(1 – r^2^)/(1 + r^2^) where r is correlation coefficient.
